# Diagnostic efficacy of monoclonal antibody based sandwich enzyme linked immunosorbent assay (ELISA) for detection of *Fasciola gigantica *excretory/secretory antigens in both serum and stool

**DOI:** 10.1186/1756-3305-4-176

**Published:** 2011-09-15

**Authors:** Zeinab A Demerdash, Tarek M Diab, Ibrahim R Aly, Salwa H Mohamed, Faten S Mahmoud, Mona K Zoheiry, Wafaa A Mansour, Mohy E Attia, Azza E El-Bassiouny

**Affiliations:** 1 Department of Immunology, Theodor Bilharz Research Institute, Giza, Egypt; 2 Department of Parasitology, Theodor Bilharz Research Institute, Giza, Egypt; 3 Department of Gastroenterology and Hepatology, Theodor Bilharz Research Institute, Giza, Egypt

**Keywords:** *Fasciola gigantica*, Monoclonal antibodies, Sandwich ELISA, Coproantigen, Seroantigen

## Abstract

**Background:**

This research was carried out to develop a reliable monoclonal antibody (MoAb)-based sandwich enzyme linked immunosorbent assay (ELISA) for the diagnosis of active *Fasciola gigantica *infection in both serum and stool for comparative purposes.

**Methods:**

From a panel of MoAbs raised against *F. gigantica *excretory/secretory antigens (ES Ags), a pair (12B/11D/3F and 10A/9D/10G) was chosen due to its high reactivity and strict specificity to *F. gigantica *antigen by indirect ELISA.

**Results:**

The two MoAbs were of the IgG_1 _and IgG_2a _subclasses, respectively. Using SDS-PAGE and EITB, the selected MoAbs recognized 83, 64, 45 and 26 kDa bands of ES Ags. The lower detection limit of ELISA assay was 3 ng/ml. In stool, the sensitivity, specificity and diagnostic efficacy of ELISA was 96%, 98.2 and 97.1%; while in serum they were 94%, 94.6% and 94.3%, respectively. Moreover, a positive correlation was found between ova count in stool of *F. gigantica *infected patients and the OD readings of ELISA in both stool and serum samples (*r *= 0.730, p < 0.01 and r = 0.608; p < 0.01, respectively).

**Conclusions:**

These data showed that the use of MoAb-based sandwich ELISA for the detection of *F. gigantica *coproantigens in stool specimens was superior to serum samples; it provides a highly efficient, non-invasive technique for the diagnosis of active *F. gigantica *infection.

## Background

*Fasciola hepatica *and *F. gigantica *are two trematode species which have an important impact on public health due to the infections they cause in humans and livestock. *F. hepatica *has a cosmopolitan distribution, mainly in temperate zones, while *F. gigantica *is found in tropical regions of Africa and Asia [1-3]. Although the majority of cases are attributed to *F. hepatica*, human infections with *F. gigantica *are also present in many countries [4-6]. In the Nile Delta of Egypt, beside the two species, a third intermediate form of *Fasciola *sp. has been identified [3] using molecular approaches [7].

Parasitological diagnosis of human fascioliasis is often unreliable and has low sensitivity, as parasite eggs are not found during the pre-patent period and shedding of parasitic eggs is intermittent [8-10]. Moreover, *Fasciola *eggs may be found in the stools of uninfected persons who have eaten raw infected liver leading to false positive diagnosis [11]. Alternatively, detection of circulating *Fasciola *antigen in both serum and stool was found to be more sensitive and specific [12]. The majority of methods based on antigen detection are applied to *F. hepatica *infection, but only few are applied to *F. gigantica *infection [13-15].

This research was carried out to establish a highly efficient MoAb-based sandwich ELISA to diagnose active *F. gigantica *infection by detecting excretory/secretory antigens (ES Ags) in both serum and stool samples of infected patients for comparative purposes.

## Methods

### Study Population

Patients admitted to Gastroenterology and Hepatology Department, Theodor Bilharz Research Institute (TBRI), who complained of abdominal pain, loss of body weight, dyspepsia, fever and diarrhea were subjected to parasitological stool examination on three consecutive days using merthiolate-iodine-formaldehyde concentration method [16]. The number of eggs per gram stool was determined by the modified Kato-thick smear technique [17]. Three groups were used; *F. gigantica *infected group where patients had the characteristic large operculated *Fasciola *eggs in their stool samples with no evidence of other parasitic infections (n = 50). Other parasites group (n = 60) included *S. mansoni *(n = 20), *S. hematobium *(n = 20) and *Hymenolepis nana *(n = 20). Control group (n = 30) were age- and sex-matched parasite-free healthy individuals.

### Stool Elute Preparation and Serum Samples Collection

Aqueous elutes of a portion of each stool specimen were prepared by adding approximately 3 parts of 0.01 M phosphate-buffered saline (PBS), pH 7.2, containing 0.05% Tween 20 (PBS/T) to 1 part of stool in a centrifuge tube [18]. The mixture was homogenized and then centrifuged at 900 × *g *for 5 min. The supernatant was aspirated and stored at -80°C until use.

Whole blood was collected from each subject and centrifuged at 760 × g at 4°C for 10 minutes and the obtained serum samples were stored at -80°C until use.

### *Fasciola *Excretory/Secretory (ES) Antigens

Livers of infected cattle were obtained from a local abattoir at Giza District, Egypt. Live intact *F. gigantica *adult worms were collected from the bile ducts and thoroughly washed at room temperature with 0.9% sodium chloride. The worms were individually incubated at 37°C in 5 ml RPMI 1640 medium, pH 7.4, supplemented with 100 U of penicillin and 100 μg of streptomycin per ml medium (Sigma Chemicals, St. Louis, USA). Following 24 h incubation, the medium was centrifuged at 1500 × *g *for 10 min at 4°C. The supernatants containing the ES Ags were aspirated [19] and filtered using filter paper No. 1 and 0.22 mm filter membrane (Millipore, Bedford, MA). The protein content was determined using Bio-Rad assay kit. Supernatants were stored at -20°C until use.

### Development of MoAbs against ES Ags

BALB/c mice were immunized with *F. gigantica *ES Ags [20]. Immune splenocytes were fused with non-secreting murine myeloma cells (P3 × 63 Ag. 8) in the presence of 43% polyethylene glycol (Sigma) [21]. Hybridomas were screened for anti-*Fasciola *antibodies by ELISA, and highly reactive hybrids were cloned by limiting dilution using a splenocyte feeder layer. Specificity was determined by indirect ELISA [22] against *S. mansoni *SEA and *E. granulosus *antigens. MoAbs showing no-cross reactivity with other parasites were selected. Hybridoma cells were injected intraperitoneally into BALB/c mice for large-scale production of MoAbs.

### Isotypic Analysis of MoAbs

Determination of MoAb isotype was done by indirect ELISA using ELISA plates coated with 20 μg/ml of *F. gigantica *ES Ags and a panel of goat anti-mouse peroxidase-conjugated immunoglobulins (IgM, IgG, IgG_1_, IgG_2a_, IgG_2b_, IgG_3 _and IgA, Sigma).

### Characterization of Target Antigen

Sodium dodecylsulphate-polyacrilamide gel electrophoresis (SDS-PAGE) and Immunoblot was carried out as described by Laemmli [23]. *F. gigantica *ES antigens was fractionated on 12.5% SDS-PAGE and either stained with Commassie brilliant blue 0.05% or transferred into nitrocellulose membrane and probed with peroxidase-conjugated IgG MoAb [24,25]. The chemical nature of MoAb-recognized epitopes was defined by testing the reactivity of target antigens by indirect ELISA before and after 20 mM sodium periodate treatment [26].

### Purification and Conjugation of MoAbs

Selected MoAbs of IgG class were purified by ammonium sulfate precipitation, dialyzed against an excess of PBS and passed through a prepacked Mono-Q-HR 5/5 column (ion exchange chromatography) [27]. The antigen detecting MoAb was conjugated to horseradish peroxidase (type VI; Sigma) [28].

### Detection of circulating *Fasciola *antigen (CFA) in both serum and stool by MoAb-based sandwich ELISA

The following sandwich-ELISA was adopted using a pair of MoAbs against ES Ags, 12B/11D/3F as antigen capturing and 10A/9D/10G as antigen detecting antibody. The optimal dilutions of MoAbs were determined by a checkerboard titration using a negative- and positive-control serum and stool samples in each plate. For each step, 100 μl/well was added unless mentioned otherwise. Polystyrene microtiter plates (Thomas Scientific, USA) were sensitized overnight at room temperature with purified 12B/11D/3F MoAb (5 μg/ml of 0.1 M carbonate buffer, pH 9.6). The plates were thoroughly washed with PBS/T (2 min/wash), and unbound sites were blocked with 200 μl/well of 2.5% fetal calf serum (Sigma) diluted in PBS/T, pH 7.4. After 2 h incubation at 37°C, the plates were emptied by suction. Undiluted serum and stool elutes were added (undiluted) and the plates were incubated for 1 h at 37°C. After thorough washing as described above, peroxidase-conjugated 10A/9D/10G MoAb (10 μg/ml of PBS/T) was added. The plates were incubated for 1 h at 37°C and washed with PBS/T. The substrate *O*-phenylenediamine dihydrochloride (Sigma) was added and the plates were incubated for 30 minutes in the dark at room temperature. The enzyme reaction was stopped with 50 μl/well of 8 N H_2_SO_4_. The absorbance at 492 nm wavelength (*A_492_*) of the plates was read using a microplate ELISA reader (Bio-Rad, Richmond CA, USA).

### Statistical analysis

Data were expressed as mean ± standard deviation. Standard diagnostic indices including sensitivity, specificity and diagnostic efficacy were calculated as described by Galen [29]. Correlations between different parameters were performed using Pearson correlation coefficient. P value greater than 0.05 was considered not significant and less than 0.01 was considered highly significant. SPSS computer program (version 12 windows) was used for data analysis.

### Ethical considerations

This study was conducted on patients admitted to Theodore Bilharz Research Institute after approval of the institutional ethical committee and obtaining an informed consent from every patient.

## Results

**Figure 1:**
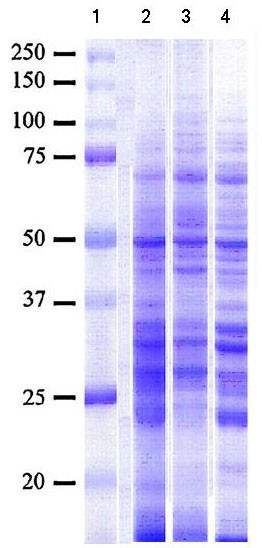
***Fasciola *ES antigens resolved from SDS-PAGE and stained by Coomassie brilliant blue 0.05%**. Lane 1: High molecular weight standard. Lanes 2, 3, 4: *Fasciola *ES antigens from three different cultures.

The SDS-PAGE analysis and Coomassie brilliant blue staining of *F. gigantica *ES Ags are shown in Figure 1. The fractionated ES Ags containing several bands ranged from 14 to 100 kDs.

### Sensitivity, Specificity and Characterization of MoAbs

**Figure 2:**
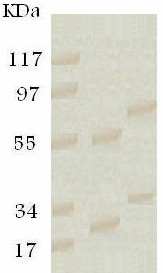
**Enzyme-linked immunoelectrotransfer blot of purified *Fasciola *ES antigen resolved on 12.5% SDS-PAGE and probed with a pair of MoAbs against ES products**. Lane 1: Low molecular weight standard. Lane 2: The 10A/9D/10G MoAb. Lane 3: The 12B/11D/3F MoAb.

From a panel of MoAbs raised against *F. gigantica *ES products, a pair (12B/11D/3F and 10A/9D/10G) was selected due to their high reactivity to *F. gigantica *antigen by indirect ELISA. Both MoAbs showed no cross-reactions with *S. mansoni, S. hematobium *or *H. nana *antigens. Isotypic analysis of the 12B/11D/3F and 10A/9D/10G MoAbs revealed that they were of IgG_1 _and IgG_2 _subclasses, respectively. The enzyme-linked immunoelectrotransfer blot (EITB) technique revealed that the two selected MoAbs recognized 83, 64, 45 and 26 kDa bands of electrophorsed ES Ag (Figure 2). The chemical nature of MoAbs-recognized epitopes was defined following 20 mM sodium periodate treatment of target antigen. Marked reduction in MoAbs' reactivity was detected (Table 1), denoting that the reactive epitopes were glycoprotein in nature.

**Table 1:** Impact of ES antigen treatment with 20 mM sodium periodate on MoAbs' reactivity by indirect ELISA (OD = 492 nm)

Target MoAbs	Before treatment	After treatment	% Reduction
*12B/11D/3F*	1.50 ± 0.15	0.71 ± 0.12	52.7%

***10A/9D/10G***	1.35 ± 0.12	0.56 ± 0.09	58.5%

Data are expressed as mean ± standard deviation or percent.

### MoAb-Sandwich ELISA

From the standard curve, the lower detection limit of ELISA assay was 3 ng/ml. The cut- off value was calculated as the mean OD reading of negative controls + 2 standard deviation of the mean. The OD readings equal to or less than cut- off value were considered negative while those readings greater than the cut off value were considered positive. The specificity of the assay was determined as the sum of results of negative control group and other parasites group.

**Figure 3:**
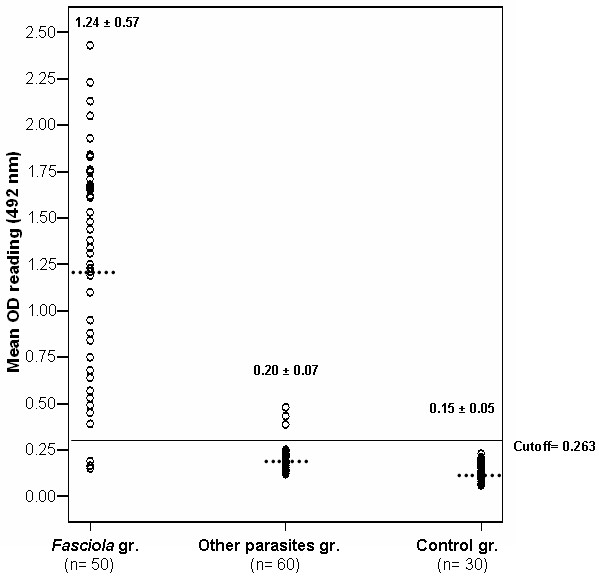
**Level of *Fasciola *ES antigen detected in serum samples of different studied groups measured by sandwich ELISA (OD reading at 492 nm)**. The horizontal line represents the cut-off value of the assay (the lower detection limit). Dotted lines represent the mean OD value.

**Figure 4:**
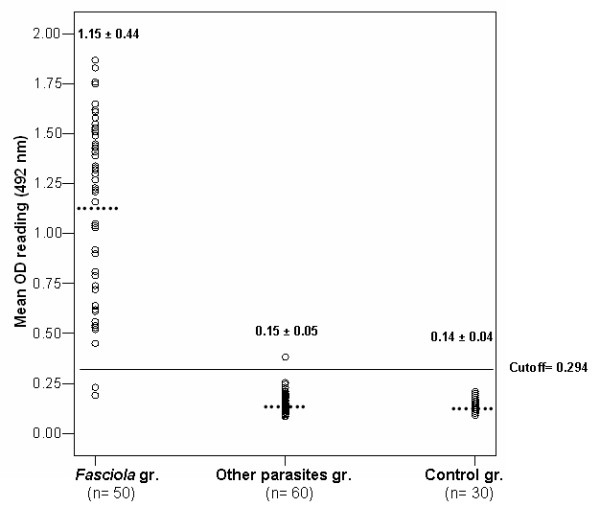
**Level of *Fasciola *ES antigen detected in stool samples of different studied groups measured by sandwich ELISA (OD reading at 492 nm)**. The horizontal line represents the cut-off value of the assay (the lower detection limit). Dotted lines represent the mean OD value.

In serum, the cut-off value was 0.263 and ES Ag was detected in 47 out of 50 *Fasciola*-infected patients and the sensitivity of the assay was 94% while in stool elutes the cut-off value was 0.294 and 48 out of 50 *Fasciola*-infected patients were positive, yielding a sensitivity of 96%. All the 20 negative controls had ES Ag levels below the cut-off value in both serum and stool samples. Furthermore, 32 out of 35 serum samples and 34 out of 35 stool elutes belonging to patients harboring other parasites had undetectable ES Ag of *Fasciola *leading to overall specificity 94.6% (52/55) and 98.2% (54/55) in serum and stool samples, respectively. The diagnostic efficacy of the assay was 94.3% and 97.1%, respectively (Figures 3, 4).

A positive correlation was found between ova count in stools of *Fasciola-*infected patients and the OD readings of ELISA in both stool and serum samples (*r *= 0.730, *p *< 0.001 and r = 0.608; p < 0.001, respectively).

## Discussion

In this study, a MoAb-based sandwich ELISA was employed for detection of circulating *F. gigantica *ES Ags in both serum and stool samples of *F. gigantica *infected patients. Identification of the target antigens recognized by anti-*Fasciola *MoAbs (12B/11D/3F and 10A/9D/10D) showed that they were glycoproteins and their MW lay in the 83, 64, 45 and 26 kDa regions. Close to our findings, Arafa *et al*. [30] produced monospecific antibodies against ES Ags of *F. gigantica *whose target antigens were recognized at 27.5, 32.5 and 55 kDa regions. They reported that cross reactivity with *Schistosoma mansoni *occurs at higher MW (110-120 kDa).

In the present study, our MoAbs were able to detect *F. gigantica *antigens level ≥ 3 ng/ml in sandwich ELISA. This could be attributed to the use of a pair of MoAbs, one as antigen capturing antibody (12B/11D/3F) which enhance the binding capacity of the other MoAb (10A/9D/10G) to the ES Ags that could help to reduce background and led to lowering the detection limit of these MoAbs. This detection limit is lower than those of Espino and Finlay [11] who detected *Fasciola *ES Ags in patients' stool at a concentration > 15 ng/ml. Also, Anuracpreeda *et al*. [31] developed a MoAb-based sandwich ELISA for detection of 28.5 kDa tegumental antigen (TA) in sera of mice experimentally infected with *F. gigantica *and found that the lower detection limit was 60 ng/ml for SE antigens, 16 ng/ml for whole body antigen, and 600 pg/ml for 28.5 kDa TA.

In the present study, the sensitivity and specificity of MoAb-based ELISA in serum was 94% and 95.6%, while in stool samples it was 96% and 98.2%, respectively. A few antigen detection assays have been developed for diagnosis of *F. gigantica *in human fluids with varied ranges of sensitivities and specificities [11,18,30]. The difference in specificities could be attributed to the complex composition of ES that makes this antigenic preparation not amenable to compare its performance in ELISA results reported from different groups. This may be due to the different protocols used to prepare ES [32-34] or to the variation in its composition when obtained from parasites derived from different hosts [35], which is not the case when a purified antigen is used [36].

In this study, the diagnostic efficacy of MoAb-based sandwich ELISA in stool (coproantigens) was superior to serum samples (97.1% vs 94.3%); this could be due to the fact that coproantigens offered several advantages, e.g., the levels of coproantigens are less affected by immune complex formation than circulating *Fasciola *antigens, coproantigens are detectable during prepatent and patent phases of infection, non-invasive and finally the nature of these antigens of being glycoprotein making the coproantigen stable under several different storage conditions which ensured its diagnostic value [11,18,31,37,38].

A positive correlation was found between ova count/gm stool of *Fasciola *infected patients and the OD readings of ELISA in both stool and serum samples. Other studies have demonstrated that coproantigens are correlated with *Fasciola *egg counts [11] and the parasite burden [31,39,40]. As the fecal egg count is presumably dependent on the number of flukes in the host, one can postulate that stool antigen level in patients infected with *Fasciola *are directly related to the number of adult parasites. Therefore, the absence of coproantigen in one of our patients may be due to a very light parasite burden and consequently undetectable levels of antigens in stools [14,18]. On the other hand, Ubeira *et al*. [32] reported that there was no correlation between number of ova/gm stool and coproantigens levels measured by ELISA.

## Conclusion

Our results provide evidence that detection of ES antigens in stool specimens improves and simplifies the diagnosis of *F. gigantica *infection in human. By using this assay, the presence of ES Ags was easily demonstrated in most of the stool specimens from patients with confirmed *F. gigantica *presenting a reliable, non-invasive diagnostic method for active infection.

## Competing interests

The authors declare that they have no competing interests.

## Authors' contributions

Both ZAD and AEE designed the study. ZAD, AEE, SHM, FSM, WAM, IRA and MKZ developed the MoAbs-based sandwich ELISA. MEA performed the clinical examinations to subjects of the study IRA and TMD collected the samples, performed the parasitological examinations and evaluated the diagnostic efficacy of the assay. TMD collected the data and performed the statistical analysis. All the authors discussed, revised, read and approved the final manuscript.
